# Interim heterogeneity changes measured using entropy texture features on T2-weighted MRI at 3.0 T are associated with pathological response to neoadjuvant chemotherapy in primary breast cancer

**DOI:** 10.1007/s00330-017-4850-8

**Published:** 2017-05-18

**Authors:** Shelley Henderson, Colin Purdie, Caroline Michie, Andrew Evans, Richard Lerski, Marilyn Johnston, Sarah Vinnicombe, Alastair M. Thompson

**Affiliations:** 1 Department of Medical Physics, Ninewells Hospital and Medical School, Dundee, UK DD1 9SY; 2 Department of Pathology, Ninewells Hospital and Medical School, Dundee, UK DD1 9SY; 3 Department of Oncology, Ninewells Hospital and Medical School, Dundee, UK DD1 9SY; 4 Division of Imaging and Technology, Ninewells Hospital and Medical School, University of Dundee, Dundee, UK DD1 9SY; 5 Department of Clinical Radiology, Ninewells Hospital and Medical School, Dundee, UK DD1 9SY; 6 Department of Breast Surgical Oncology, University of Texas MD Anderson Cancer Centre, Houston, TX 77030 USA

**Keywords:** Magnetic resonance imaging, Breast cancer, Neoadjuvant chemotherapy, Lesion hetereogeneity, Pathogical response

## Abstract

**Objectives:**

To investigate whether interim changes in hetereogeneity (measured using entropy features) on MRI were associated with pathological residual cancer burden (RCB) at final surgery in patients receiving neoadjuvant chemotherapy (NAC) for primary breast cancer.

**Methods:**

This was a retrospective study of 88 consenting women (age: 30–79 years). Scanning was performed on a 3.0 T MRI scanner prior to NAC (baseline) and after 2–3 cycles of treatment (interim). Entropy was derived from the grey-level co-occurrence matrix, on slice-matched baseline/interim T2-weighted images. Response, assessed using RCB score on surgically resected specimens, was compared statistically with entropy/heterogeneity changes and ROC analysis performed. Association of pCR within each tumour immunophenotype was evaluated.

**Results:**

Mean entropy percent differences between examinations, by response category, were: pCR: 32.8%, RCB-I: 10.5%, RCB-II: 9.7% and RCB-III: 3.0%. Association of ultimate pCR with coarse entropy changes between baseline/interim MRI across all lesions yielded 85.2% accuracy (area under ROC curve: 0.845). Excellent sensitivity/specificity was obtained for pCR prediction within each immunophenotype: ER+: 100%/100%; HER2+: 83.3%/95.7%, TNBC: 87.5%/80.0%.

**Conclusions:**

Lesion T2 heterogeneity changes are associated with response to NAC using RCB scores, particularly for pCR, and can be useful across all immunophenotypes with good diagnostic accuracy.

***Key Points*:**

• *Texture analysis provides a means of measuring lesion heterogeneity on MRI images*.

• *Heterogeneity changes between baseline*/*interim MRI can be linked with ultimate pathological response*.

• *Heterogeneity changes give good diagnostic accuracy of pCR response across all immunophenotypes*.

• *Percentage reduction in heterogeneity is associated with pCR with good accuracy and NPV*.

## Introduction

Neoadjuvant chemotherapy (NAC) for primary breast cancer is clinically useful to downstage locally advanced breast cancer and/or reduce the extent of surgery from mastectomy to breast conservation. Response to NAC varies by subtype of breast cancer and chemotherapy regimen [1, 2] but prediction of response remains clinically challenging [3, 4]. A pathological complete response (pCR) to NAC is intrinsically linked with survival outcomes, particularly in triple negative and hormone receptor positive disease [5–7] and, in the future, where confidence is high that a pCR has been achieved, post-treatment surgery might not be required [8, 9]. Therefore, early identification of such patients becomes crucial for improving and personalising patient management. Conversely, identification of patients unlikely to respond well early in treatment may allow modification to the cytotoxic regimen or dose, although the benefits of such a strategy would need to be proven in prospective trials [10].

Breast magnetic resonance imaging (MRI) is increasingly used to monitor patients during NAC therapy, allowing repeated examinations, with dynamic contrast-enhanced imaging (DCE) providing a measure of tumour vascularity. However, breast MRI can under- or overestimate the amount of residual tumour in around 30% of women [11], and the accuracy of response prediction is dependent on immunohistochemical tumour subtype [12].

Quantitative imaging biomarkers play an important role in repeat imaging studies [13], particularly when comparative changes in response to a therapy require quantification. To this end, a number of MRI metrics have been explored, including MRI DCE parameters [14], volume changes [3, 15] and diffusion changes [16].

The application of textural analysis (TA) to medical imaging studies shows promise. TA statistically models spatial distributions of pixel grey-levels in order to classify or segment the data [17] and has been used successfully in neuroimaging for tissue differentiation and classification [18, 19]. For breast MRI, TA has been used to differentiate between malignant and benign lesions [20, 21] and in discrimination between breast cancer subtypes [22–24].

Recently, heterogeneity, as assessed using TA, has found a role in the diagnosis, characterization [25] and treatment response assessment of various cancer types [26, 27], with initial reports suggesting an association with patient survival [28]. Preliminary work utilising TA [29] indicates that heterogeneity changes in response to NAC may be seen using T2-weighted imaging, as well as on DCE-MRI. T2-weighted imaging has the advantage that signal intensity is directly related to underlying morphology (via intracellular and extracellular/extravascular space) and can provide useful information in cases where contrast material cannot be administered. T2-weighted images are also less susceptible to degradation (e.g. movement) and are likely to be easier to standardise across multiple sites. As previous studies using TA have been relatively small, we explored application of these principles in a larger patient cohort, correlating with a recognised, validated measure of pathological response to NAC for breast cancer- the residual cancer burden score (RCB) [30]. Patients with a pathologic complete response (pCR) have favourable survival rates compared with those having minimal, moderate or extensive residual disease (RCB-I/RCB-II/RCB-III) [30].

This study sought to determine if changes in lesion heterogeneity could be linked with response to NAC, as measured using ultimate pathological outcome and assess whether the response association was dependent on cancer immunophenotype. Measurement repeatability was also considered, as is essential when evaluating a potential quantitative tool.

## Methods

### Subjects

**Fig. 1:**
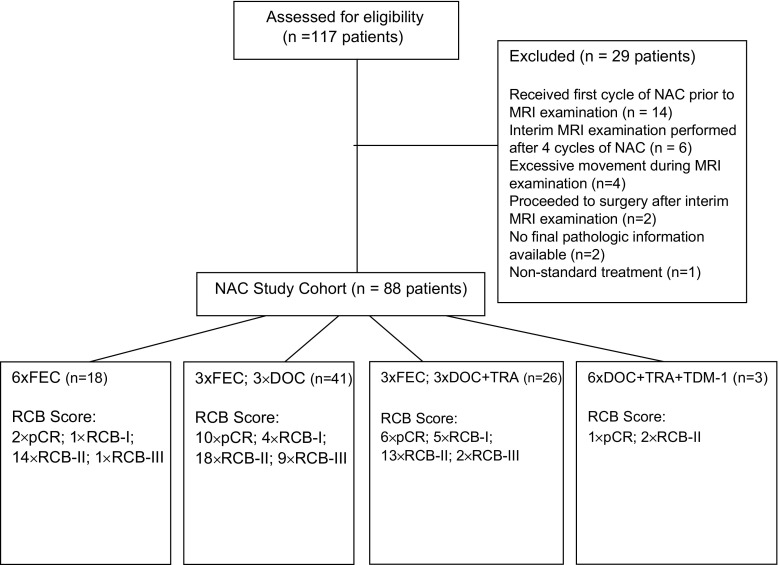
Diagram of recruitment and study population including neoadjuvant chemotherapy (NAC) regime and response as measured using residual cancer burden (RCB) score. *FEC* fluorouracil, epirubicin (75) and cyclophosphamide, *DOC* docetaxel, *TRA* trastuzumab, *TDM*-*1* trastuzumab emtansine

This study comprised women with biopsy-proven primary breast cancer, scheduled for NAC prescribed either to facilitate breast conservation or render a locally advanced breast cancer operable, according to national guidelines [31]. All women gave written consent for use of their images for research purposes. Informed consent and ethical approval was waived for this retrospective analysis of anonymised data collected in the course of routine clinical care. From 117 consecutive women scanned over a 19-month period, 28 patients were excluded from the study due to MRI timing, incomplete data, poor image quality or non-standard management (Fig. 1). The remaining 88 patients (age range: 30–79, mean age 50.7 years) comprised the study cohort, who underwent three routine MRI examinations: after diagnosis but prior to NAC, mid treatment and following completion of NAC, prior to surgery.

Patient and tumour characteristics are summarised in Table 1.

**Table 1:** Patient and tumour characteristics

	Patients (total n = 88)
Inclusion period	November 2012–April 2016
Mean age in years (range)	51 (30–79)
Histological type	
Invasive ductal	84
Invasive lobular	2
Other	2
Grade	
Grade 2	19
Grade 3	69
Pre-treatment MRI size	
< 20 mm	4
20–40 mm	54
> 40 mm	30
Immunohistochemistry	
ER+	26
HER2+	29
TN	33
Neoadjuvant chemotherapy	
6 × FEC	18
3 × FEC; 3 × DOC	41
3 × FEC; 3 × DOC + taxane	29

In 88 patients, 26 cancers were oestrogen receptor (ER) positive (Allred score >3), HER2 negative; 29 HER2 positive (immunohistochemistry 3+ or immunohistochemistry 2+ and fluorescence *in situ* hybridisation (FISH) amplified); 33 triple negative breast cancer (TNBC) (Allred score <3 and HER2 0, 1+ or 2+ with FISH non-amplified). The HER2 positive group included women who were hormone-receptor positive (n = 22) and negative (n = 7). Eighteen patients received six cycles of FEC (fluorouracil, epirubicin and cyclophosphamide); 41 women received three cycles of FEC followed by three cycles of docetaxel. All 29 patients with HER2 positive disease had three cycles of FEC followed by three cycles of docetaxel and trastuzumab (n = 26) or docetaxel with trastuzumab-emtansine (TDM1) (n = 3).

### MR imaging

Patients were scanned prior to NAC, after NAC cycle 2 (n = 16) or 3 (n = 72) and prior to surgery to assess final imaging response. At interim MRI examination, all patients had received only FEC treatment. The median time between final MRI examination and surgical resection was 28 days (range: 6–57 days).

All MR examinations were carried out using a 32-channel 3.0 Tesla (T) Siemens Magnetom Trio scanner (Erlangen, Germany) and a 7-channel breast coil. A T2-weighted sequence was acquired using a turbo-spin echo sequence (TR/TE/α = 8,000 ms/86 ms/150°, field of view = 340 × 340 mm, matrix = 320 × 320, voxel size 1.1 mm^3^), with parallel imaging factor × 2 and turbo factor × 21. The rest of the examination consisted of a T1 non-fat-saturated acquisition followed by the DCE acquisition.

DICOM images were anonymised and exported off-line to perform TA.

### Texture analysis

**Fig. 2:**
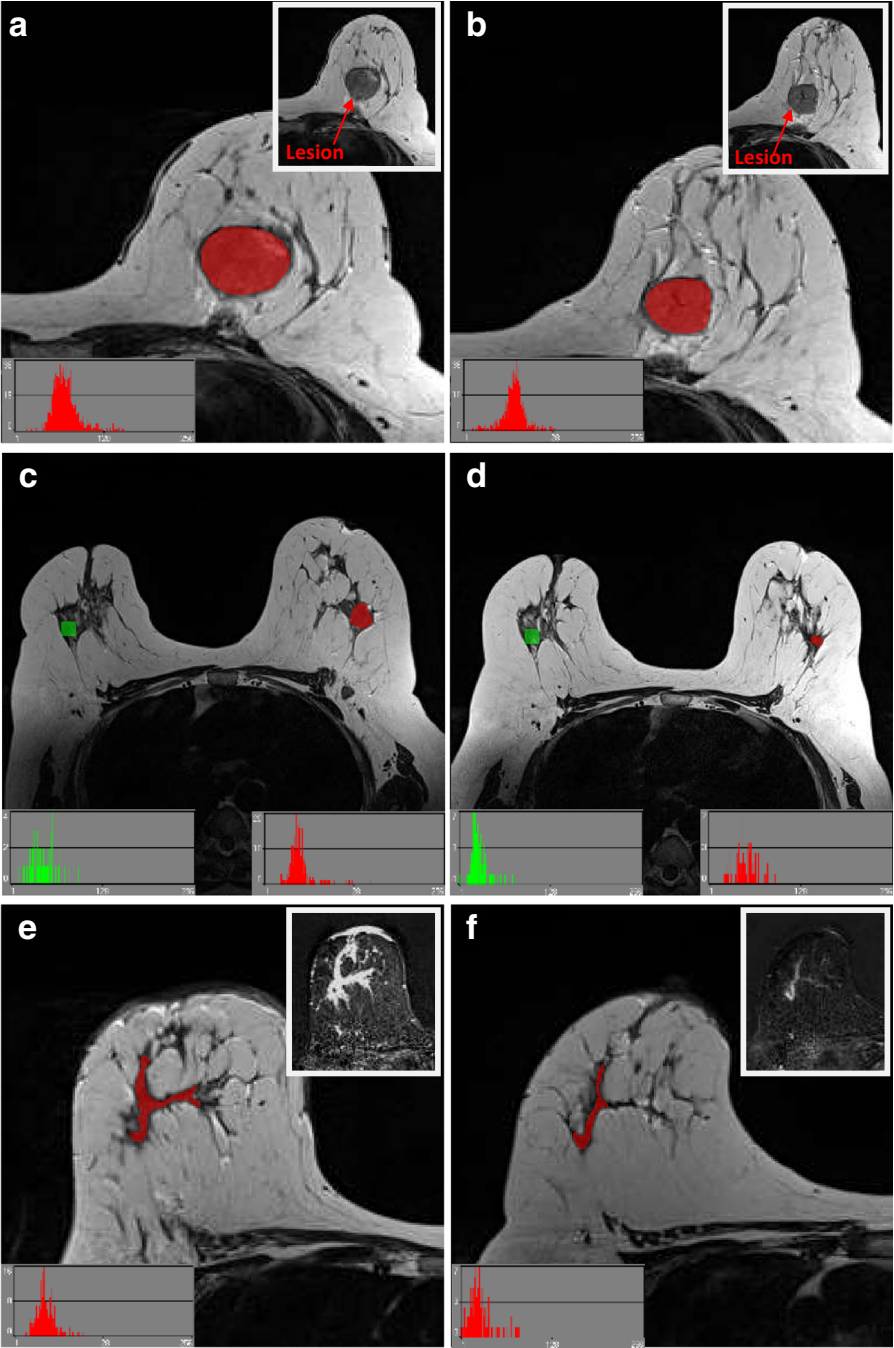
Region of interest (ROIs) (red) drawn for texture analysis on two patients, with slices matched between baseline (left column) and interim (right column) examinations. Insert images highlight lesion locations without ROI overlay. Image (**a**) and (**b**) are from slice-matched T2-weighted images from a woman who had a RCB-III at final pathology, while (**c**) and (**d**) are from slice-matched images in a patient that ultimately achieved a pCR at end of treatment. Images (**e**) and (**f**) highlight the importance of correlation with the subtracted dynamic contrast-enhanced imaging (DCE) images, particularly in the case of non-mass enhancement, as shown

Texture analysis was performed using MaZda version 4.7 [32] on baseline and interim T2-weighted images. Post-contrast subtracted images from both examinations of each patient were considered side-by-side to identify maximum lesion diameter on each; subsequently these slices were matched to the appropriate slice on the T2 acquisition for region of interest (ROI) positioning on comparable slices (see Fig. 2). All image analysis was performed blinded to patient outcome, clinical and pathology information.

T2-weighted images were magnified to visualise the tumour border and an ROI drawn around the entire tumour on the imaging slice demonstrating maximum lesion diameter, with no marker clip present. Histograms were visualised to ensure no fat was erroneously present within the ROI (see Fig. 2). A 10 × 10 pixel ROI was placed in healthy, normal contralateral breast parenchyma to exclude systematic inter-scan differences (as shown in Fig. 2(c,d)). These were placed in comparable regions of breast parenchyma at baseline and interim to ensure consistency and care was taken to exclude presence of vessels within the ROI. Data was rescaled to 6 bits/pixel and the image histogram normalized to within 3 standard deviations of the mean to minimise brightness and contrast variations. Entropy features, representing heterogeneity, were derived from the grey-level co-occurrence matrix [33], as these are reported in the literature as most appropriate for cancer imaging [29]. Inter-pixel distances of n = 2 and n = 5 were calculated to depict fine and coarse texture. Raw feature values were exported for statistical analysis.

Intra- and interobserver error was calculated based on a subset of 20 baseline images that were analysed twice by SAH (8 years TA experience), with a 1-month interval, and once by RAL (30+ years TA experience).

### Assessment of response

RCB score [30], was calculated by a specialist breast pathologist (CP) using final pathology of resected specimen, based on tumour bed dimensions, cellularity and axillary node burden and reported in terms of the RCB index [30]. Patients were categorised in terms of whether they achieved a pCR post treatment, or had residual disease (RCB-I, RCB-II, RCB-III).

### Statistics

All statistics were calculated using SPSS (v21; IBM Corporation, New York, NY, USA) with p < 0.05 considered significant, and p < 0.001 highly significant.

Intra- and interobserver measurement error of entropy features were calculated using coefficients of variation (CoVs) and Pearson’s intraclass correclation coefficient (ICC).

Data was not normally distributed therefore non-parametric tests were used throughout. Absolute baseline and interim entropy values were compared using Wilcoxon tests in both normal and lesion tissue, and percentage changes between examinations assessed using Kruskal-Wallis tests (across all response categories) and Mann-Whitney U-tests (between response categories).

Receiver operating characteristic (ROC) curves were generated and areas under the curve (AUCs) calculated. Optimal thresholds were derived using Youden’s index, and resultant sensitivity, specificity, accuracy, and positive and negative predictive values (PPVs and NPVs) calculated.

## Results

### Patient cohort

Of 88 patients analysed, 107 lesions were identified; however, TA was restricted to the largest, index lesion in each case. At final pathological examination, 16 patients were categorized as pCR, 12 as RCB-I, 43 as RCB-II and 17 as RCB-III (Fig. 1).

### Reproducibility

Intra-observer ICC values demonstrated a high correlation for entropy features (ICC = 0.813, p < 0.001) with corresponding CoV value of 5.4%. Inter-observer ICC values demonstrated similar correlations, with entropy ICC = 0.790 (p < 0.001) and a slightly poorer CoV value of 8.0%.

### RCB response

Mean baseline ROI size was 795 pixels (range: 202–1,995), corresponding to a physical size of 962 mm^2^ (244–2,414 mm^2^); interim ROI size was 454 pixels (77–1,561) with physical size 549 mm^2^ (93–1,889 mm^2^). There were no significant differences in baseline TA features between response categories (p > 0.866; Kruskal-Wallis).

**Fig. 3:**
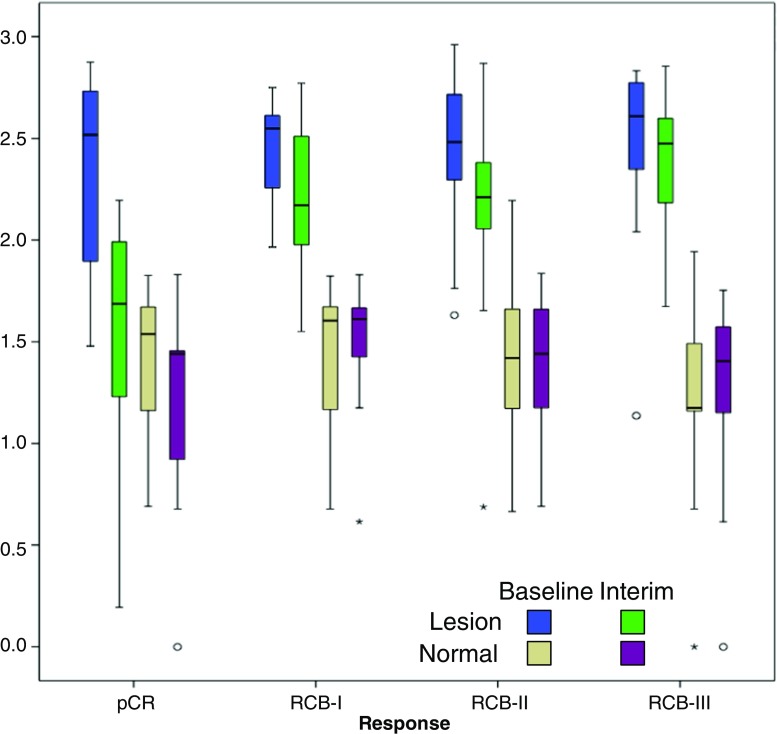
Coarse entropy features at baseline and interim examinations in both normal tissue and lesion in each response category. Within normal tissue there were no significant differences between visits for any of the residual cancer burden (RCB) response categories (p > 0.419; Wilcoxon test), while within the lesion entropy features demonstrate a reduction in lesion heterogeneity at the interim examination

No significant differences were found within normal parenchyma between baseline and interim examinations (p > 0.419; Wilcoxon test) or in pair-wise comparisons of difference in entropy features between response groups (p > 0.166; Mann-Whitney U-test) (Fig. 3), suggesting no random or systematic inter-scan temporal differences.

**Fig. 4:**
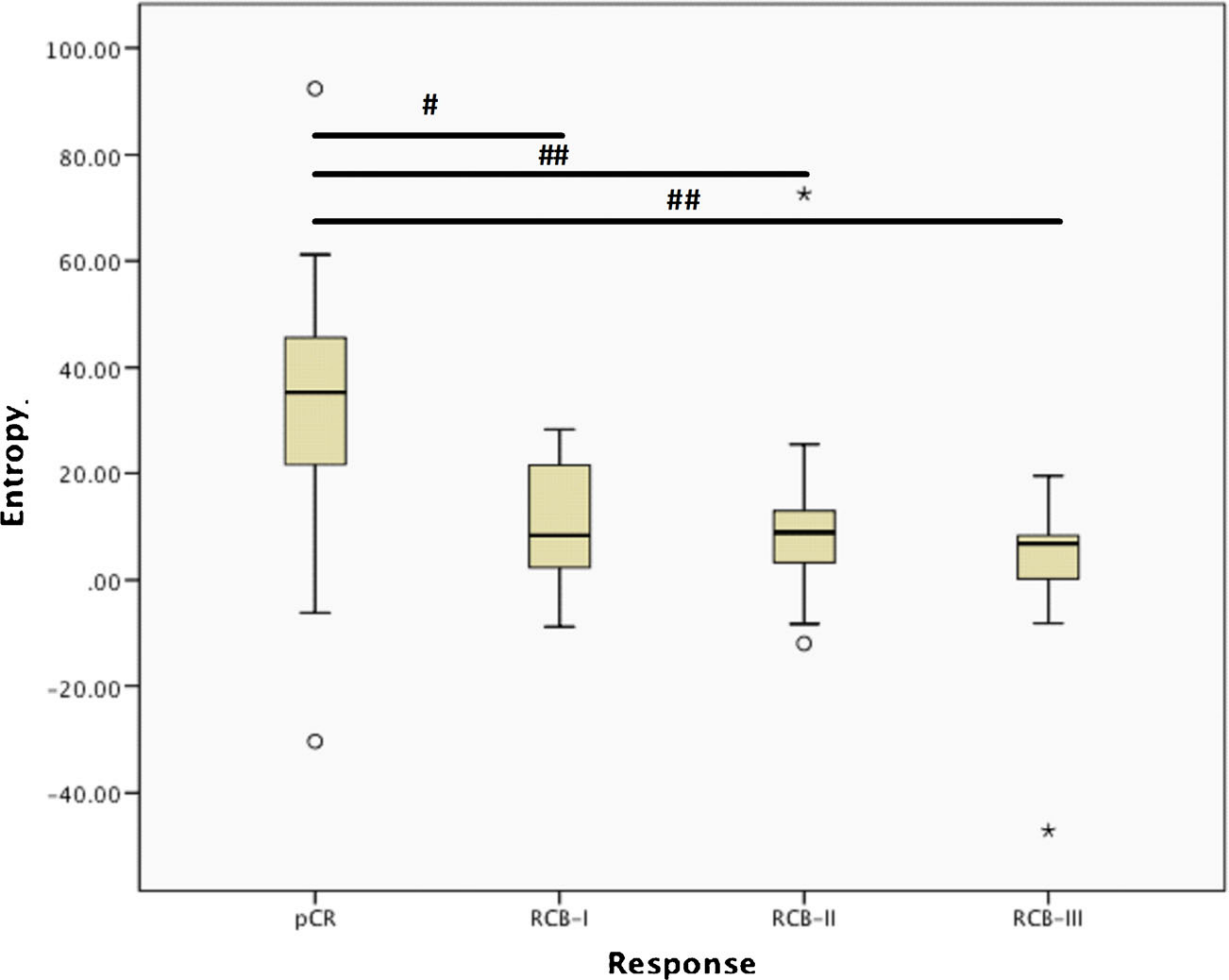
Percentage reduction in coarse entropy features between baseline and interim examinations for each response category as measured using the residual cancer burden (RCB) score. Statistical comparisons are indicated (^#^p < 0.05, ^##^p < 0.001; Mann-Whitney U-test)

Patients with pCR, RCB-I or RCB-II scores had decreases in fine and coarse entropy features between baseline and interim examinations (p < 0.006; Wilcoxon test), indicating lesion image heterogeneity reduction. No significant differences were found for RCB-III patients (p > 0.397; Wilcoxon test) (Table 2, Fig. 3). Median decreases in coarse entropy between baseline and interim MRI were pCR: 32.8%, RCB-I: 10.5%, RCB-II 9.7% and RCB-III: 3.0% (Fig. 4). For both entropy features, patients with pCR had a significantly greater reduction than those patients with residual disease (p < 0.014; Mann-Whitney U-test).

**Table 2:** Median differences between baseline and interim examinations for coarse and fine entropy in each residual cancer burden (RCB) response category, and Wilcoxon statistical tests (*indicates significance at p < 0.05 level)

		Entropy	
		fine	coarse
pCR	Median (range)	0.485 (-0.064–1.477)	0.862 (-0.447–2.360)
		**p** = 0.002*	**p** < 0.001*
RCB-I	Median (range)	0.200 (-0.236–0.584)	0.206 (-0.225–0.780)
		**p** = **0.060**	**p** = **0.061**
RCB-II	Median (range)	0.143 (-0.279–1.058)	0.218 (-0.231–1.819)
		**p** = 0.006*	**p** < 0.001*
RCB-III	Median (range)	0.118 (-0.569–0.414)	0.164 (-0.536–0.510)
		**p** = **0.557**	**p** = **0.397**

*pCR* pathological complete response

**Fig. 5:**
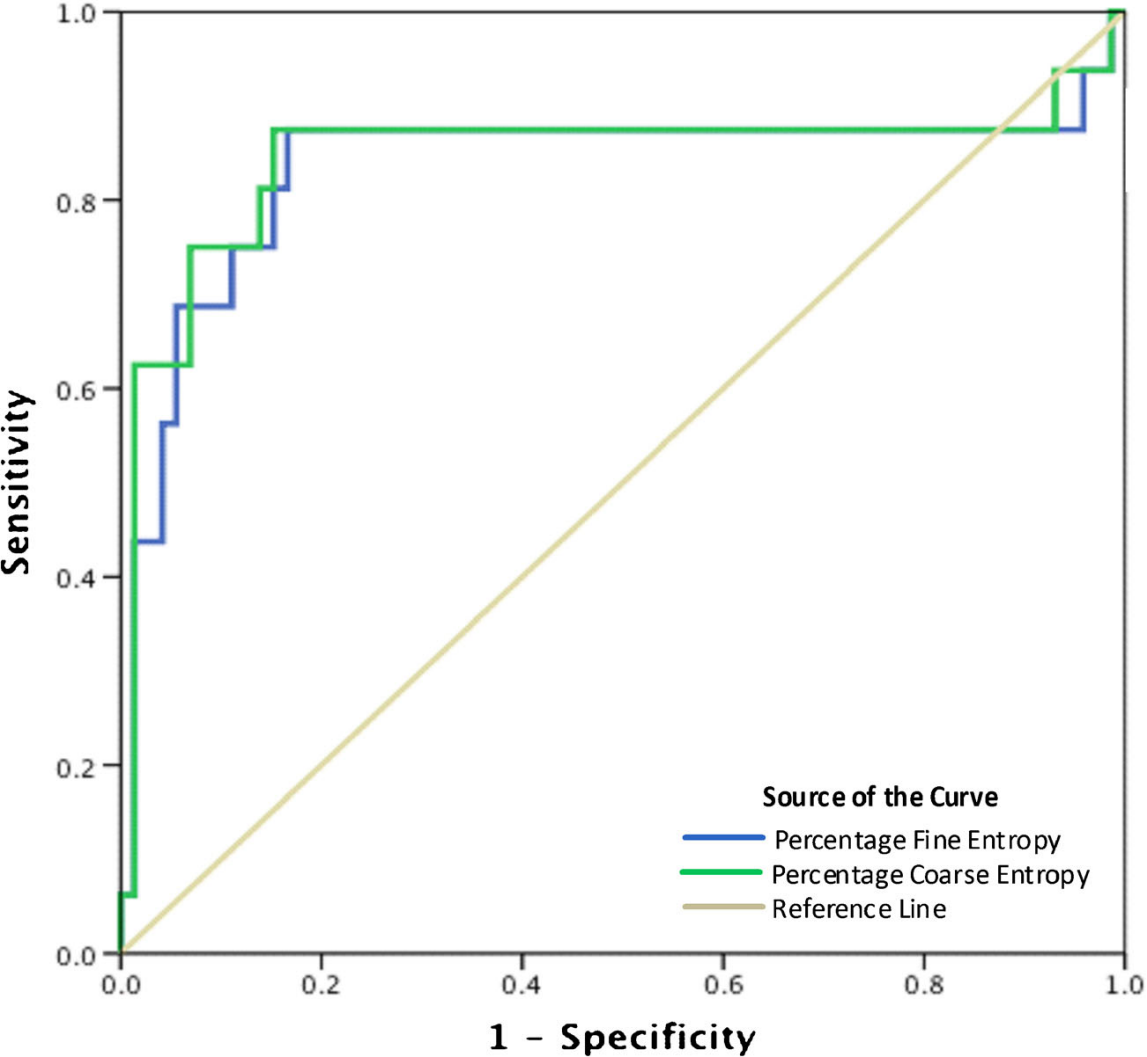
Receiver operating characteristic (ROC) curve for fine and coarse entropy features in the classification of pathological complete response (pCR). Areas under the curve are 0.834 and 0.845, respectively

The ROC curve for fine and coarse entropy is shown in Fig. 5, with calculated AUC for pCR classification of 0.834 using fine features and 0.845 using coarse features. Optimal thresholds for pCR identification were derived as 11% (fine entropy) and 20% (coarse entropy) using Youden’s index. Resulting sensitivities, specificities, accuracies, NPVs and PPVs are shown in Table 3.

**Table 3:** Test performance results in the identification of pathological complete responders (pCRs) when using reduction in entropy features – both coarse and fine

	Entropy coarse	Entropy fine
	(20% reduction)	(11% reduction)
AUROC	0.845	0.834
Sensitivity	87.5%	87.5%
Specificity	84.7%	81.9%
Accuracy	85.2%	83.0%
PPV	56.0%	51.8%
NPV	96.8%	96.7%

*AUROC* area under the receiver operating characteristic curve, *PPV* positive-predictive value, *NPV* negative-predictive value

Tumours were then considered in terms of immunophenotype. There were significant differences between pCR and residual disease groups when considered across all immunophenotypes (ER+: p < 0.001; HER2+: p < 0.007; TNBC: p < 0.001; Mann-Whitney U-test). Median percentage changes in coarse entropy categories for ER+, HER2+ and TNBC (Table 4) confirmed reduction in lesion image heterogeneity was associated with pathological response as assessed by RCB with changes in heterogeneity being greatest in the pCR group with ER+ tumours. Patients with TNBC who ultimately achieved a pCR had a lower reduction in heterogeneity with a median 23% reduction indicating a likely pCR post-NAC. Optimal thresholds were derived using Youden’s index as ER: 28%, HER2+: 30% and TNBC: 18%. These resulted in good diagnostic characteristics, as presented in Table 5.

**Table 4:** Median percentage changes in lesion image heterogeneity as assessed using coarse entropy features for ER+, HER2+ and TNBC in each response category (n = number of cancers per group)

	ER+ (n = 26)	HER2+ (n = 29)	TNBC (n = 33)
pCR	42.9% (2)	35.3% (6)	22.8% (8)
RCB-I	8.4% (3)	8.4% (5)	14.6% (4)
RCB-II	8.1% (10)	4.2% (16)	11.1% (14)
RCB-III	6.8% (11)	9.5% (2)	4.5% (7)

TNBC triple negative breast cancer, ER oestrogen receptor, *RCB* residual cancer burden

**Table 5:** Test performance results in the identification of pathological complete responders (pCRs) when using reduction in coarse entropy features for ER+, HER2+ and triple negative breast cancers (TNBCs)

	ER+ (n = 26)	HER2+ (n = 29)	TNBC (n = 33)
pCR	42.9% (2)	35.3% (6)	22.8% (8)
RCB-I	8.4% (3)	8.4% (5)	14.6% (4)
RCB-II	8.1% (10)	4.2% (16)	11.1% (14)
RCB-III	6.8% (11)	9.5% (2)	4.5% (7)

^a^ threshold derived using Youdens index

*PPV* positive predictive value, *NPV* negative predictive value

## Discussion

Neoadjuvant chemotherapy is increasingly used in breast cancer management, particularly for TNBC and HER2+ disease, and a pCR is directly related to overall survival [5–7, 34]. Image analysis carries potential to assess response during therapy, prior to surgical resection, but most studies using breast MRI have focused on changes in DCE or apparent diffusion coefficient [16, 35]. This study considered changes in T2-weighted MRI entropy features (representing heterogeneity) during NAC and correlated with pathological RCB score post-treatment. We demonstrate a significant association between heterogeneity changes and RCB, and thus, indirectly, with clinical outcome [30, 34]. Importantly, the technique has performed well for each immunophenotype and thus further investigation is of real clinical importance.

Absolute differences in fine entropy features between baseline and interim examinations dichotomised data into two statistically distinct and clinically important groups; pCR and RCB-I, II and III (residual disease). ROC analysis demonstrates that a threshold reduction of 20% in coarse entropy resulted in an association of patients who would ultimately have a pCR, with an accuracy of 85.2% (AUC 0.845). Importantly, these thresholds result in a very good NPV for pCR (Tables 3 and 5), therefore clinicians could potentially identify at interim MRI patients unlikely to achieve a pCR on therapy completion, with over 95% accuracy.

Based on our results, coarse entropy features perform better than fine features, with greater differences between baseline and interim MRI. This larger threshold in heterogeneity reduction for pCR leads to improved diagnostic accuracy (Table 3, Fig. 5). Fine and coarse entropy use has been reported elsewhere [29] and relates to the scale at which structural features are enhanced within textural findings. Previous hypotheses suggest fine features relate to parenchyma while coarser features represent underlying vasculature; however these are based on CT images and have not been validated in MR imaging.

No significant differences were found in heterogeneity of the contralateral breast, lending weight to findings being attributable to therapeutic effects. While chemotherapy-induced changes have been reported within normal breast tissue [36], most likely relating to vascular damage [37], these are less likely to affect T2-weighted images.

There is scant literature concerning repeatability of TA in clinical cohorts; however, our intra-observer measurement error of CoV = 5.4% is in line with Carballido-Gamio et al. who report 2.8–6.6% intra-observer repeatability in entropy measures of knee cartilage [38] and Mathias et al. who quote 1.1% within the spinal cord [39]. We can find no studies that consider inter-observer repeatability measures, however, estimate our CoV at 8%. With estimated error below 10%, this could potentially mean the technique could be comparable across multicentre studies.

The reduction in entropy has previously been reported [29]; however, we have extended this work to consider the association of pCR versus residual disease at the end of treatment. While this was a pilot study, our patient numbers were larger than comparable studies [29, 40, 41], an accepted measurement of pathological response was used [30] and the important consideration of immunophenotype was included.

Although baseline prediction of ultimate response to NAC would have real clinical importance, we found no link in our cohort of patients. Reports in the literature on baseline heterogeneity response prediction are contradictory; with Ahmed et al. reporting increased pre-treatment entropy in association with poor NAC response [42], whereas studies by Teruel [40] and Michoux [41] suggest the opposite. It should be noted, however, that none of these studies consider immunophenotype, which is particularly relevant in the NAC response setting.

Prediction of pCR at interim MRI has been widely investigated, and two recent papers have considered various metrics for this purpose. O’Flynn et al. evaluated a range of parameters including volumes, pharmacokinetics and enhancement ratios. They found the best pCR predictor was volumetric changes, which resulted in an area under the ROC curve of 0.773 and sensitivity/specificity of 71.4%/76.9% [43]. Li et al. focused on optimising enhancement thresholds for calculating tumour volumes and obtained a value of AUC = 0.73 [44]. This group also considered immunophenotype with a good AUC of 0.77 (ER+/HER2-), 0.75 (HER2+) and 0.85 (TNBC). Our study has shown higher diagnostic accuracies, and it would therefore be of great interest to evaluate the two combined techniques in future studies.

Given the association with RCB score, TA has the potential to support trial development where, after an imaging assessment of complete response to NAC, no resection would be performed, only percutaneous sampling of the tumour bed and radiotherapy treatment to the breast. Such a concept, while not new [9, 45], may have a resurgence of interest given the increasing efficacy of NAC, if a sufficiently accurate non-invasive modality for monitoring, such as MRI TA, were available.

Texture analysis has wide application in cancer imaging for differentiation, classification and treatment monitoring [22–29, 40, 42]. Spatial resolution is key in generalisability across scanners [46], and while the technique is computationally demanding, both in analysis and interpretation, incorporation into commercial, validated post-processing software should be possible. Presently, different software applications result in entropy values with different orders of magnitude and therefore utility of absolute entropy features in diagnosis is unlikely to come to fruition until standardisation occurs. However, the most useful, and perhaps clinically relevant, utility is in comparative studies – be that comparison of tissue types or in follow-up studies, such as treatment monitoring. Currently, the precise correlate of TA with underlying anatomical or pathological structures is unknown and other groups are working on linking imaging heterogeneity with underlying histopathological and genetic heterogeneity [24].

While most TA of breast images have been performed on post-contrast images, these are subject to movement, and registration algorithms can introduce artificial texture into the images. In a recent comparative study, TA of T2 images was found to be more discriminatory between clinical response groups in patients undergoing NAC than DCE-MRI [29]. The underlying mechanism for signal intensity distributions in DCE-MRI and T2-weighted imaging is entirely different. Signal intensity in DCE-MRI is derived from contrast agent distribution and vascular leakage, while T2-weighted imaging is predominantly influenced by intracellular and extracellular/extravascular space and is therefore more likely to directly reflect tumour morphology.

The heterogeneous nature of cancer subtypes included in this study led to the use different chemotherapy regimes, which reflects clinical reality. However, interim analysis was performed on patients who had only undergone FEC treatment. Another potential weakness of the study is that interim scans were performed after two or three cycles of NAC, yet despite this variability we were still able to show statistically significant differences between response categories. Finally, there is a potential for a slight mismatch in analysed imaging slice between baseline and interim MRI examination. This mismatch is likely to be small, especially in relation to tumour size changes, imaging slice thickness and the use of anatomical landmarks and marker clips for localisation of ROI positioning.

In conclusion, lesion heterogeneity changes are associated with response to NAC using RCB scores, and could be useful in identification of patients likely to achieve a pCR, across all immunophenotypes. These data suggest changes in MRI entropy features may provide a clinically useful early indication of response to NAC and warrants further investigation and consideration in multivariate analyses for prediction of pathological results.
